# Editorial

**Published:** 1839-09-28

**Authors:** 


THE MEDICAL EXAMINER.

PHILADELPHIA, SEPTEMBER 28, 1839.

### THE ALBANY MEDICAL COLLEGE AND THE THOMSONIANS.

An outcry has been raised against the Faculty of the Albany Medical College, for not closing their doors upon the Thomsonian students. This outcry has, we think, proceeded, from a mistaken view of the interests and dignity of the profession. Science has nothing to apprehend from contact with ignorance, nor can the issue be doubtful, when truth and falsehood meet face to face. The Albany College is about to fight the battle of the profession against quackery with the best weapon that can be employed—*education.* It has acted most sagaciously in bringing the Thomsonian forces under the battery of "anatomy, surgery, chemistry, and physiology." Such a surrender on the part of the enemy, we consider the virtual abandonment, or at least a pledge of the entire modification, of their system of practice. Whatever be their views, if they persevere in their course, the downfall of the heresy must follow.

It appears that the Albany Faculty have simply permitted the Thomsonian students, upon paying the matriculation fee, to take what tickets they please—in fact, have granted them no exclusive privilege at all. Such, we are convinced, would be the course of the Medical Schools generally.

